# Clinical Characteristics and Outcome of Neuronal Surface Antibody-Mediated Autoimmune Encephalitis Patients in a National Cohort

**DOI:** 10.3389/fneur.2021.611597

**Published:** 2021-03-09

**Authors:** Zsófia Hayden, Beáta Bóné, Gergely Orsi, Monika Szots, Ferenc Nagy, Tünde Csépány, Zsolt Mezei, Cecília Rajda, Diána Simon, József Najbauer, Zsolt Illes, Timea Berki

**Affiliations:** ^1^Department of Immunology and Biotechnology, Clinical Center, University of Pécs Medical School, Pécs, Hungary; ^2^Department of Neurology, University of Pécs, Pécs, Hungary; ^3^MTA-PTE Clinical Neuroscience MR Research Group, Pécs, Hungary; ^4^Department of Neurosurgery, Clinical Centre, University of Pécs Medical School, Pécs, Hungary; ^5^Department of Neurology, Somogy County Kaposi Mór University Teaching Hospital, Kaposvár, Hungary; ^6^Department of Neurology, University of Debrecen, Debrecen, Hungary; ^7^Department of Neurology, Semmelweis University, Budapest, Hungary; ^8^Department of Neurology, University of Szeged, Szeged, Hungary; ^9^Department of Neurology, Odense University Hospital, Odense, Denmark; ^10^Institute of Clinical Research, BRIDGE, University of Southern Denmark, Odense, Denmark

**Keywords:** autoimmune encephalitis, neuronal surface antibody, clinical characteristics, immunotherapy, prognosis

## Abstract

**Background:** In our previous single-center study of autoimmune encephalitis (AE) related autoantibody test results we found positivity in 60 patients out of 1,034 with suspected AE from 2012 through 2018 as part of a Hungarian nationwide program. In our current multicenter retrospective study, we analyzed the clinical characteristics and outcome of AE patients with positive neuronal cell surface autoantibody test results.

**Methods:** A standard online questionnaire was used to collect demographic and clinical characteristics, laboratory and imaging data, therapy and prognosis of 30 definitive AE patients in four major clinical centers of the region.

**Results:** In our study, 19 patients were positive for anti-NMDAR (63%), 6 patients (20%) for anti-LGI1, 3 patients for anti-GABABR (10%) and 3 patients for anti-Caspr2 (10%) autoantibodies. Most common prodromal symptoms were fever or flu-like symptoms (10/30, 33%). Main clinical features included psychiatric symptoms (83%), epileptic seizures (73%) and memory loss (50%). 19 patients (63%) presented with signs of central nervous system (CNS) inflammation, which occurred more frequently in elder individuals (*p* = 0.024), although no significant differences were observed in sex, tumor association, time to diagnosis, prognosis and immunotherapy compared to AE patients without CNS inflammatory markers. Anti-NMDAR encephalitis patients were in more severe condition at the disease onset (*p* = 0.028), although no significant correlation between mRS score, age, sex and immunotherapy was found. 27% of patients (*n* = 8) with associated tumors had worse outcome (*p* = 0.045) than patients without tumor. In most cases, immunotherapy led to clinical improvement of AE patients (80%) who achieved a good outcome (mRS ≤ 2; median follow-up 33 months).

**Conclusion:** Our study confirms previous publications describing characteristics of AE patients, however, differences were observed in anti-NMDAR encephalitis that showed no association with ovarian teratoma and occurred more frequently among young males. One-third of AE patients lacked signs of inflammation in both CSF and brain MRI, which emphasizes the importance of clinical symptoms and autoantibody testing in diagnostic workflow for early introduction of immunotherapy, which can lead to favorable outcome in AE patients.

## Introduction

Autoimmune encephalitis (AE) is increasingly recognized as one of the most frequent causes of non-infectious encephalitis (1, 2). In AE, the most common autoantibodies against neuronal cell surface receptors and synaptic proteins include those against NMDAR (N-methyl-D-aspartate receptor), GABABR (γ-aminobutyric acid receptor-B), AMPAR (α-amino-3-hydroxy-5-methyl-4-isoxazolepropionic acid receptor) and against proteins associated with the VGKC (voltage-gated potassium channel complex), such as LGI1 (leucine-rich, glioma inactivated 1) and Caspr2 (contactin-associated protein-like 2) (3, 4). AE may present with a wide spectrum of clinical symptoms, such as behavioral and psychiatric disorders, cognitive impairment, changes in the level of consciousness, seizures and movement disorders (5, 6). Besides clinical features suggestive of AE, accurate diagnosis relies on the detection of characteristic autoantibodies in the serum and/or cerebrospinal fluid (CSF), accompanied with auxiliary examinations, such as structural magnetic resonance imaging (MRI) and electroencephalography (EEG) (7). The diagnosis of AE may be complicated as results of cerebral imaging and CSF analysis are often unremarkable, especially in the elderly with autoantibodies against LGI1 and Caspr2 (8, 9). In AE, early introduction of proper therapy is associated with a more favorable outcome, emphasizing the importance of early diagnosis based on clinical symptoms and paraclinical tests at the time of presentation. In our previous single-center study of patients with suspected AE, we found positive autoantibody test results in 60 out of 1,034 patients from 2012 through 2018 as part of a Hungarian nationwide program (10). In that study, long-term (1–5 years) autoantibody testing was conducted in 30 patients with positive autoantibody results, of which 17 remained positive at different time points. The aim of the present retrospective study was to characterize the clinical features and outcome of AE, based on the results of laboratory and paraclinical tests, in a multicenter cohort of Hungarian patients, involving four major clinical centers of the region to collect data of 35 patients with AE-related autoantibody positivity.

## Materials and Methods

### Patient Inclusion

In our current study, we retrospectively identified 35 patients with positive neuronal cell surface autoantibody (NMDAR, LGI1, GABABR, Caspr2). Patients were selected based on our previous observational cohort study of patients who tested positive for at least one neuronal cell surface autoantibody in sera, CSF or both in sera and CSF at the Department of Immunology and Biotechnology, Clinical Center, University of Pécs Medical School, Pécs, Hungary, from January 2012 until December 2019 (10). Of the total 35 patients, 24 were tested in CSF-serum pairs (68.6%), 9 for serum only (25.7%) and 2 for CSF only (5.7%). Five patients with positive antibody results were excluded from further clinical analysis because of alternative diagnosis. Two patients with anti-Caspr2 positivity detected in sera but not in CSF, and one patient with anti-NMDAR positivity in CSF but not in serum had a final diagnosis of stroke (of which the latter patient died). One patient with anti-NMDAR positivity found in sera but not in CSF, and one patient with anti-Caspr2 positivity detected only in sera (CSF was not available) had a final diagnosis of multiple sclerosis. Patients with positive neuronal cell surface autoantibody tests enrolled in this study fulfilled the criteria for definite AE defined by Graus et al. (7): (1) subacute onset (rapid progression <3 months) of working memory deficits (short-term memory loss), altered mental status, or psychiatric symptoms, and (2) new focal central nervous system (CNS) findings, seizures not explained by a previously known seizure disorder, CSF pleocytosis (white blood cell count > 5 cells/mm^3^), or MRI features suggestive of encephalitis, and (3) reasonable exclusion of alternative causes. All anti-NMDAR autoantibody positive patients included in the study had ≥2 positive results during confirmatory tests (Euroimmun, The anti-Glutamate Receptor (type NMDA) IIFT kit, FC112d1005-51).

### Neuronal Cell Surface Antibody Detection

The antibody panel included anti-NMDAR, anti-GABABR, anti-AMPAR1, anti-AMPAR2, anti-LGI1 and anti-Caspr2. AE-related antibody testing was performed in the routine laboratory, using indirect immunofluorescence (IIFT) BIOCHIP assay with HEK293 cells expressing the genes of the six different proteins (Euroimmun, Autoimmune Encephalitis Mosaic 1, FA 112d-1003-1). The assay was optimized following the guideline included in the Manufacturer's Instruction. The anti-Glutamate Receptor (type NMDA) IIFT kit with BIOCHIP slides containing fields of NMDAR expressing and control HEK293 cells was used as a confirmatory test (Euroimmun, The anti-Glutamate Receptor (type NMDA) IIFT kit, FC112d1005-51) in case of samples positive or equivocal for anti-NMDAR autoantibodies. For histological localization of autoantibody positivity a rat brain biochip was used for indirect immunofluorescence imaging (Euroimmun, IIFT: Glutamate Receptor Mosaic 3, FA 111m-1003-3).

### Data Collection and Analyses

Clinical data were collected using an online questionnaire in collaboration with neurologists specialized in neuroimmunology from four clinical centers in Hungary. Questions about demographics, prodromal symptoms, clinical features, CSF findings, EEG and brain MRI descriptions, therapy and prognosis were included in the questionnaire.

We used the modified Rankin scale (mRS) to measure neurological outcome in AE patients (11–14). The mRS score was determined at onset, at the time of the worst status of the patient, and at the last visit. The mRS score of 0-2 at the last visit was considered as good outcome and > 2 as poor outcome (13). Complete recovery was assessed in AE patients with mRS score of 0 at the last visit following treatment (median: 33 months; range: 1–77) (12). Relapse was defined as the new onset or worsening of symptoms, after at least 2 months of improvement or stabilization (15).

### Statistical Methods

Statistical evaluation was performed with the SPSS IBM version 26 (IBM, Armonk, NY, USA). Categorical variables were described as percentages and numerical variables were described as medians and ranges. Mann-Whitney U test for continuous variables and Fisher's exact test for categorical variables were used as appropriate. *P*-values < 0.05 were considered statistically significant.

### Standard Protocol Approvals, Registrations, and Patient Consents

Clinical samples were obtained with patients' informed consent. The study was approved by the Ethics Committee of the Medical Research Council of Hungary (number of approval: 49709-2/2019/EKU).

## Results

### Demographic Features and Characteristics of AE Patients

We identified and collected detailed clinical data of 30 patients with the diagnosis of definite AE. Of the 30 patients, autoantibodies were tested in CSF-serum pairs in 66.7%, only in serum in 26.7% and only in CSF in 6.7% of patients (Table 1). The most common antibody was anti-NMDAR (19/30, 63.3%), followed by anti-LGI1 (6/30, 20%), anti-GABABR (3/30, 10%) and anti-Caspr2 (3/30, 10%). One patient showed positivity for both anti-LGI1 and anti-Caspr2 antibodies. In the anti-NMDAR patient group, autoantibodies were detected in sera and CSF simultaneously in 31.6%, only in CSF in 36.8% and only in sera in 31.6% of patients. All anti-NMDAR autoantibody positive patients had ≥2 positive results during confirmatory tests (Euroimmun, The anti-Glutamate Receptor (type NMDA) IIFT kit, FC112d1005-51). In the anti-LGI1 and in the anti-Caspr2 positive patients, in all cases autoantibodies were present only in sera, but not in the CSF with the exception of one patient with anti-LGI1 and anti-Caspr2 antibody positivity detected in both sera and CSF. In all anti-GABABR positive patients, we detected antibodies both in sera and CSF (Table 1). The group of 30 patients with definite AE, included 19 men (63.3%) and 11 women (36.7%) with a median age of 39.3 years (range: 1–75 years). Different sex ratios and median age were observed in the different AE types (Table 1).

**Table 1:** Demographic data and tested sample types in AE patients.

	**Autoimmune encephalitis**	**anti-NMDAR**	**anti-LGI1**	**anti-GABABR**	**anti-Caspr2**
	**(*n* = 30)**	**(*n* = 19)**	**(*n* = 6)**	**(*n* = 3)**	**(*n* = 3)**
**Age (range)**	39.3 (1-75)	32.5 (1-75)	46.8 (3-65)	47 (16-67)	47.7 (3–72)
**Sex (M/F)**	19:11	11:8	5:1	2:1	2:1
Cured Complete recovery (n, %)^*^	20 (66.7%)	14 (73.7%)	4 (66.7%)	0	3 (100%)
**Death (n, %)**	3 (10%)	1 (5.3%)	1 (16.7%)	1 (33.3%)	0
**Relapse (n, %)**	1 (3.3%)	1 (5.3%)	0	0	0
***Tested sample types***					
CSF-serum pairs (n, %)	20 (66.7%)	12 (63.2%)	4 (66.7%)	3 (100%)	2 (66.7%)
Only CSF (n, %)	2 (6.7%)	2 (10.5%)	-	-	-
Only sera (n, %)	8 (26.7%)	5 (26.3%)	2 (33.3%)	-	1 (33.3%)

* *Complete recovery was defined as the proportion of AE patients with mRS score of 0 at the last visit following treatment (median: 33 months; range: 1–77)*.

*M, male; F, female; NMDAR, N-methyl-D-aspartate receptor; LGI1, leucine-rich, glioma inactivated 1; GABABR, γ-aminobutyric acid receptor-B; Caspr2, contactin-associated protein-like 2, CSF, cerebrospinal fluid*.

### Clinical Features of AE Patients

Median time to diagnosis (onset of clinical symptoms until positive autoantibody result) was 2 months (range 1–53 months). The most common prodromal symptoms were fever or flu-like symptoms (10/30, 33.3%), presenting mostly in anti-NMDAR encephalitis (8/19, 42.1%). One anti-NMDAR patient had herpes simplex virus (HSV) encephalitis confirmed by positive PCR in the CSF, and NMDAR encephalitis developed about a month after discharge from the hospital. Four additional patients with anti-NMDAR encephalitis had HSV IgM in the serum (Supplementary Table 1).

**Figure 1:**
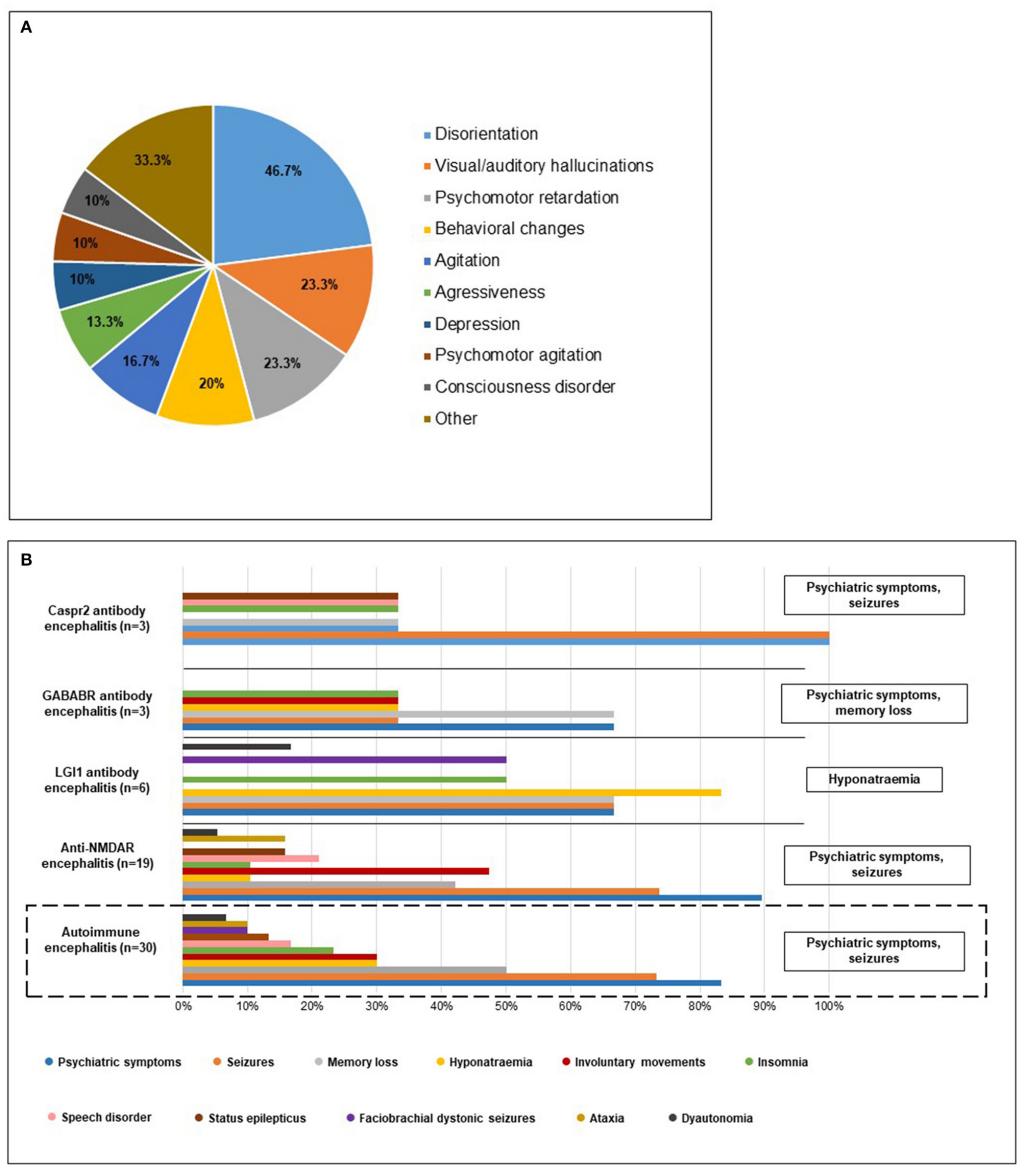
**(A)** Psychiatric symptoms of AE patients. The diagram shows the distribution of psychiatric symptoms among the 30 AE patients in our cohort. Most common symptoms included disorientation, visual/auditory hallucinations, psychomotor retardation and behavioral changes. Frequency of rare psychiatric symptoms such as anxiety, cognitive deficit, catatonia, apathy, hypersexuality, and mutism are included in “Other.” **(B)** Characteristic clinical symptoms of AE patients. The bars show the frequency of different clinical features of AE patients. The diagram at the bottom shows that psychiatric symptoms and seizures were the most common in the 30 AE patients in our cohort. The other diagrams indicate distribution of clinical features separately in different AE types. Different distribution of clinical symptoms was observed in anti-LGI1 and anti-GABABR patients: in LGI1 encephalitis hyponatraemia and in GABABR encephalitis besides psychiatric symptoms memory loss was among the most common symptoms.

The most common initial presentations were psychiatric symptoms before the onset of neurologic dysfunction (17/30, 56.7%) (Supplementary Table 2): 11 patients with anti-NMDAR, 3 patients with LGI1, 2 patients with GABABR and 2 patients with Caspr2. During the disease course, psychiatric symptoms were altogether present in 25/30 (83.3%) of AE patients. In our cohort, psychiatric symptoms included the presence of one or more of the following: anxiety, apathy, aggressiveness, agitation, depression, hypersexuality, disorientation, visual/auditory hallucinations, behavioral changes, cognitive deficit, psychomotor retardation/agitation, catatonia, consciousness disorder, and mutism. The most common psychiatric symptom was disorientation (14/30), followed by visual/auditory hallucinations (7/30), psychomotor retardation (7/30) and behavioral changes (6/30) (Figure 1A). In pediatric cases (age <10 years: Patient 13, 14, 18, and 30), psychiatric symptoms included the presence of one or more of the following: disorientation, behavioral changes and catatonia.

Other clinical features included seizures (22/30), memory loss (15/30), insomnia (7/30), speech disorders such as dysarthria or aphasia (5/30), status epilepticus (4/30) and ataxia (3/30). Piloerection, cerebellar symptoms, neuropathy and skin rashes occurred in singular cases, respectively. Involuntary movements, such as dyskinesia, dystonia or choreoathetosis were the most common in NMDAR encephalitis (9/19). Orofacial dyskinesia was present in 6/19 anti-NMDAR positive patients accompanied with hand dyskinesia in two patients. Dystonia occurred in two patients with NMDAR encephalitis, and choreoathetosis was observed in one NMDAR patient. Dysautonomia was present in one anti-NMDAR positive patient. Hyponatraemia (5/6) and faciobrachial dystonic seizures (3/6) were the most frequent in the LGI1 patient group. Clinical features of the different types of AE are summarized in Figure 1B.

Regarding associated tumors, eight cases (26.7%) were observed: in three anti-NMDAR, three anti-GABABR, one anti-LGI1 and one anti-Caspr2 patients. Five patients were male (62.5%) and median age was 62.5 years (range: 16–72 years). The most common tumor type was lung carcinoma (large cell lung carcinoma in one NMDAR patient and small cell lung carcinoma in two GABABR patient). In most cases (5/8, 62.5%), positive neuronal autoantibody test result preceded the detection of associated tumor. Median time from autoantibody positivity until detection of tumor was 15 months (Supplementary Table 3).

### Auxiliary Examinations of AE Patients

**Figure 2:**
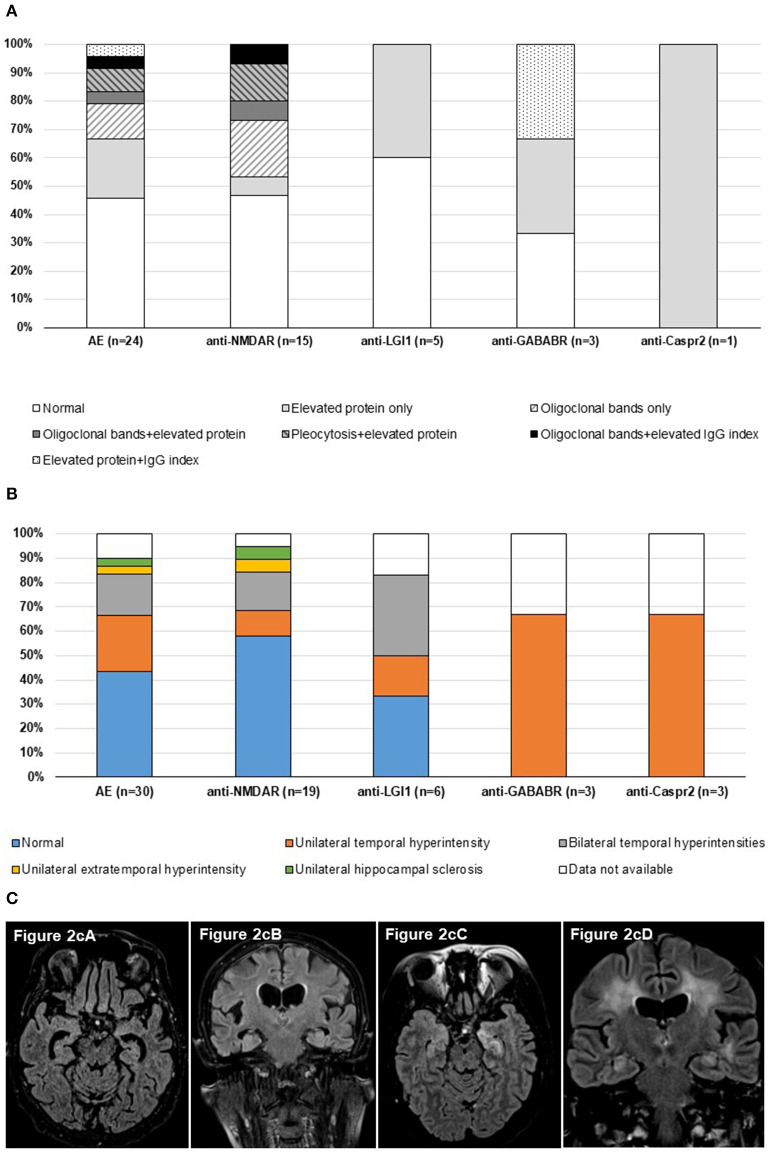
**(A)** Composition of cerebrospinal fluid in AE patients. The bars demonstrate CSF analyses result of 24 AE patients from our cohort. At least one abnormal CSF finding was found in 13 AE patients, most commonly in anti-NMDAR and anti-GABABR patients. Pleocytosis and OCB were detected only in NMDAR encephalitis. **(B)** Brain MRI findings in AE patients. The diagram shows brain MRI result of 27 AE patients from our cohort. In 14 AE patients, abnormalities were found, mainly in the form of unilateral or bilateral temporal hyperintensities. **(C)** Axial and coronal FLAIR brain images from patient 8 (cA, cB) and patient 24 (cC, cD). Patient 8 was a 75-year-old female patient, who had anti-NMDAR autoantibody positivity detected in CSF and had hyperintensity and contrast enhancement within bilateral medial temporal lobes (cA-axial 3D FLAIR, **c**B-coronal T2 FLAIR). Patient 24 was a 50-year-old female patient, who had anti-LGI1 autoantibody positivity detected in serum and had bilateral limbic encephalitis, dominant in the left temporal lobe, both on axial (cC) and coronal T2 FLAIR images (cD).

CSF was analyzed in 80% (24/30) of AE patients (Figure 2A). Abnormal CSF findings, such as pleocytosis (white blood cell count <5 cells/mm^3^), the presence of oligoclonal bands (OCB), increased protein level (> 450 mg/L) and/or elevated IgG index (> 0.65) were observed in 13/24 of the tested AE patients. The majority of patients with anti-NMDAR (8/15) and anti-GABABR (2/3) had abnormal CSF results. Pleocytosis (2/15, 13.3%) and OCB (5/15, 33.3%) were detected exclusively in NMDAR patients. Elevated IgG index was found in one anti-NMDAR and one anti-GABABR patient. Altogether, increased protein level was the most common abnormal CSF finding (9/24, 37.5%), accounting for all abnormal CSF cases in both the LGI1 and Caspr2 patient groups (Figure 2A).

We were able to evaluate EEG data in 29 patients. EEG abnormalities were observed in 14/29 patients (Supplementary Table 4). The most common abnormal EEG findings were focal slowing (6/30) and interictal epileptiform discharges (4/30) (Supplementary Figure 1).

Brain MRI examination was evaluated in 27 patients. Abnormalities on brain MRI were observed in 14/27 patients (Supplementary Table 5). Abnormal brain MRI results were mainly unilateral or bilateral lesions in the insula/hippocampus (13/30) (Figures 2B,C). In 7/14 (50%) of AE patients with abnormal brain MRI results, follow-up brain MRI examinations were conducted (median: 24 months, range: 2–37 months), which revealed hippocampal sclerosis or cerebral atrophy in three anti-LGI1 and two anti-NMDAR positive patients (5/7, 71.4% of patients with follow-up brain MRI result).

### Treatment and Outcomes

In 24/30 of AE patients, first-line immunotherapy was applied. Most commonly methylprednisolone pulse was used in combination with plasmapheresis (12/30). Other therapy included only the use of methylprednisolone (4/30) or in combination with intravenous immunoglobulin (5/30). Combination of all three types of immunotherapy was administered in 4/30 cases: three anti-NMDAR patients (mRS 5 at admission) presenting with psychiatric symptoms, seizures and involuntary movements, and one anti-LGI1 patient (mRS 3 at admission) with FBDS, hyponatraemia, dysautonomia and insomnia required such combination. All four AE patients had good outcome at the last visit (mRS 0). In one case, plasmapheresis was applied alone. Most patients (22/30, 73.3%) responded to the first-line therapy. Second-line therapy was introduced with azathioprine or rituximab or with the combination of the two in four cases with anti-NMDAR encephalitis presenting with severe disability during admission (mRS ≥ 4) and with no significant improvement to first-line therapy (Supplementary Table 6). Azathioprin and/or rituximab was continued as maintenance therapy in three out of these four AE patients, due to residual symptoms (memory loss, psychiatric symptoms). Antiepileptics were used exclusively in 6/30 (20%) of AE patients, presenting predominantly with seizures and showing good outcome at the last visit (mRS median: 0; range: 0–1).

Introduction of immunotherapy was followed by at least one repeated neuronal cell surface autoantibody test in 20/30 (66.7%) of AE patients (median: 8.3 months, range: 1–47). Antibody positivity persisted in 11/20 (55%) of repeatedly tested patients (seven anti-NMDAR, three anti-GABABR and one anti-LGI1 encephalitis patients). The median hospital stay of AE patients was 23 days. Patients were severely impaired on admission, with a median mRS score of 4 (range 2–5). Most AE patients showed significant improvement after treatment and 25/30 (83.3%) achieved a good outcome (mRS ≤ 2). Median follow-up duration was 33 months (range 1–77) (Table 2). Five patients had a mRS ≥ 3 at the last visit, including one patient with anti-NMDAR and one patient with anti-GABABR who died of consequences of associated lung tumor and one patient with anti-LGI1 who died of deep vein thrombosis. One patient with anti-GABABR and small cell lung cancer was bedridden and required continuous nursing. One patient with anti-NMDAR had persistent cognitive deficit and memory loss, requiring some help as being unable to perform all previous activities. In our cohort, AE patients with associated tumor (*n* = 8) had a significantly higher mRS score at the time of the last visit (median: 2.5, range: 0–6) compared to AE patients without tumor (*n* = 22; mRS at the last visit median: 0, range: 0–3) (*p* = 0.045).

**Table 2:** Comparison of AE patients regarding the presence of CNS inflammation and associated autoantibody type.

**Variables**	**With CNS inflammation**	**Without CNS inflammation**	**anti-NMDAR encephalitis**	**anti-LGI1, anti-GABABR, anti-Caspr2 encephalitis**
	**(*n* = 19)**	**(*n* = 11)**	**(*n* = 19)**	**(*n* = 11)**
Age (range)	54 (16-75)	17 (1-70)	32.5 (1-75)	58 (3-72)
Male (*n*, %)	14 (73.7%)	5 (45.5%)	11 (57.9%)	8 (72.7%)
Tumor (*n*, %)	6 (31.6%)	2 (18.9%)	3 (15.8%)	5 (45.5%)
Diagnostic delay (median, months)	3	1	1	5
mRS at diagnosis (median)	3	5	5	3
mRS at last visit (median)	0	0	0	0
**Status at Last Visit**				
mRS 0–2 (*n*, %)	15 (78.9%)	10 (90.9%)	17 (89.5%)	8 (72.7%)
mRS 3-6 (*n*, %)	4 (21.1%)	1 (9.1%)	2 (10.5%)	3 (27.3%)
**Immunotherapy**				
Only steroid (*n*, %)	3 (15.8%)	1 (9.1%)	3 (15.8%)	1 (9.1%)
Steroid+IVIG (*n*, %)	0	2 (18.9%)	1 (5.3%)	1 (9.1%)
Steroid+PE (*n*, %)	10 (52.6%)	2 (18.9%)	5 26.3%)	5 (45.5%)
Steroid+IVIG+PE (*n*, %)	1 (5.3%)	3 (27.3%)	1 (5.3%)	1 (9.1%)
Second-line therapy (*n*, %)	2 (10.5%)	2 (18.9%)	4 (21.1%)	0

*CNS inflammation was defined as the presence of at least one of the CSF inflammatory markers, such as pleocytosis (white blood cell count <5cells/mm3), oligoclonal bands, elevated protein or IgG index and/or brain MRI lesions suggestive of encephalitis (mesial temporal T2 hypersignal or signs of demyelination)*.

*CNS, central nervous system; NMDAR, N-methyl-D-aspartate receptor; LGI1, leucine-rich, glioma inactivated 1; GABABR, γ-aminobutyric acid receptor-B; Caspr2, contactin-associated protein-like 2; mRS, modified Rankin scale score; IVIG, intravenous immunoglobulin; PE, plasmapheresis*.

The rate of complete recovery in AE patients (follow-up median: 33 months; range: 1–77 months), showed favorable prognosis (20/30), with the highest rate in the anti-Caspr2 patient group. Relapses were uncommon in AE patients (1/30): only one male patient relapsed, whose anti-NMDAR positivity persisted in CSF during repeated autoantibody tests (follow-up: 47 months; number of autoantibody tests: 4). Death occurred in 3/30 of AE patients (Table 1).

### Comparison of AE Patients Based on Signs of CNS Inflammation and Associated Autoantibody Types

We analyzed the cohort based on the presence of CNS inflammatory markers, i.e. CSF inflammatory changes and brain MRI abnormalities. We defined CNS inflammation as the presence of at least one of the CSF inflammatory markers, such as pleocytosis (white blood cell count > 5 cells/mm^3^), oligoclonal bands, elevated protein or IgG index and/or brain MRI lesions suggestive of encephalitis (mesial temporal T2 signal hyperintensity or signs of demyelination). CNS inflammation was present in 19/30 patients (63.3%): both CSF and MRI abnormalities were detected in 7/30 patients (23.3%), 6/30 patients (20%) had brain MRI lesions suggestive of encephalitis without altered CSF findings, and 6/30 patients (20%) had signs of inflammation in CSF, but no abnormal brain MRI findings. In the remaining 11/30 cases, no changes indicating CNS inflammation were detected. Table 2 shows a comparison of clinical data of AE patients presenting with or without CNS inflammatory markers. In the patient group with inflammatory changes, age of onset was significantly higher (*p* = 0.024). No significant differences in sex, frequency of tumor association, time to diagnosis, prognosis and type of immunotherapy were observed between AE patients with and without CNS inflammatory markers.

We also analyzed AE patients based on the type of neuronal autoantibodies (Table 2). Patients were classified as anti-NMDAR positive and positive for other AE-related antibodies (anti-LGI1, anti-GABABR, anti-Caspr2). NMDAR encephalitis patients (*n* = 19) were in more severe condition at the onset of the disease with significantly higher mRS score at the time of diagnosis (median: 5, range: 2-5) compared to LGI1, GABABR and Caspr2 encephalitis (*n* = 11; mRS score at the diagnosis median: 3, range: 2–5) (*p* = 0.028). A trend of longer time to diagnosis was observed in the non-NMDAR patient group compared to NMDAR positive patients (*p* = 0.063). However, in two LGI1-encephalitis cases, final diagnosis of AE was retrospectively confirmed after the introduction of cell-based assay in our laboratory in 2012. No significant differences were found in sex, frequency of tumor association, prognosis and type of immunotherapy between anti-NMDAR positive AE patients and patients positive for other AE-related antibodies (anti-LGI1, anti-GABABR, anti-Caspr2).

## Discussion

We aimed to examine the characteristics of AE including clinical, laboratory, MRI features and outcome of AE in a Hungarian cohort. In accordance with previous publications of other series (6, 16–18), the most common autoimmune encephalitis type was anti-NMDAR encephalitis, followed by anti-LGI1 encephalitis (3, 19, 20). The study by Bien et al. (21) reported 576 antibody-positive patients during testing of 10,919 patients for a broad panel of neural antibodies, including onconeural and neuronal cell surface autoantibodies. Our previous study (10) included results of 60 neuronal cell surface autoantibody positive patients (anti-NMDAR, anti-LGI1, anti-GABABR, anti-Caspr2, anti-AMPAR1, 2) and our recent study exclusively included both serologically (neuronal cell surface antibody positive, but not onconeural autoantibody positive) and clinically positive definite AE patients. The distinct results in autoantibody frequencies detected in the two laboratories, may be due to the exclusive inclusion of neuronal cell surface autoantibody positive AE patients in our study, making it impossible to compare accurately the data of autoantibody frequencies. In our cohort, the sex ratio was close to equal, with NMDAR encephalitis occurring slightly more frequently in men than women. Similar sex ratio was observed in a cohort of Chinese AE patients (12), although, published data claims that anti-NMDAR encephalitis affects predominantly young women (median age, 21 years) (3, 22). Dalmau et al. (3) reported male predominance in LGI1, GABABR and Caspr2 encephalitis, which was also observed in our cohort, although, the median age of our AE patients were 45–50 years compared to median values of 60–65 years in previous publications (3, 23).

In 70–86% of anti-NMDAR encephalitis patients, prodromal symptoms, such as headache, fever, nausea and vomiting were present (19, 24, 25). In our cohort, HSV infection occurred in one patient, who had secondary NMDAR encephalitis one month after HSV encephalitis. Previous publications (26) have reported HSV infection as being the most common viral trigger in AE patients and a possible trigger of anti-NMDAR encephalitis. The precise mechanism of HSV triggered AE is not clearly defined. Molecular mimicry or breakdown of immunological tolerance may be in the background (27). It has been stated that the most common symptoms of AE are psychiatric symptoms, seizures, involuntary movements, memory loss and sleep disorders (23). Besides these characteristic features, some rare manifestations, such as piloerection also emphasized in a systematic review and related to LGI1 encephalitis (28), cerebellar symptoms, neuropathy and skin rashes were also observed in our cohort. Previous publications have reported of FBDS as a typical symptom of anti-LGI1 encephalitis, often occurring a few weeks before onset of cognitive deficit in 26–71% of patients (20). In a retrospective study in the UK (29), 77% (20/26) of anti-LGI1 encephalitis patients experienced FBDS prior to the development of limbic encephalitis and found that early immunotherapy for FBDS might prevent progression to cognitive impairment. A prospective study of nine patients with anti-LGI1 encephalitis also revealed the beneficial effect of early immunotherapy (30). In our cohort of AE patients, FBDS was exclusively present in the LGI1 encephalitis patients with a lower prevalence (50%) compared to previous publications. Besides FBDS, hyponatraemia was also predominant in this group.

Examination of CSF has an important role in diagnosis, as the presence of pleocytosis is included in the diagnostic criteria for AE (7), although AE associated with autoantibodies against LGI1 and Caspr2 sometimes lack signs of inflammation in the CSF. The study of Hébert et al. (31) reported prevalence of CSF inflammatory markers, including elevated white blood cell count, elevated protein concentration and OCB in 95 patients with early active AE. Similarly to our study, where 46% of AE patients had normal CSF results upon testing, in the cohort of Hébert et al. 44% of AE patients lacked CSF pleocytosis, 27% of patients, in addition to the lack of CSF pleocytosis, had normal protein concentration in the CSF and 14% of AE patients, besides the mentioned two CSF parameters, lacked OCB. In a retrospective study of anti-LGI1 encephalitis patients (32), CSF pleocytosis was identified in 23% of patients. Meanwhile, NMDAR and GABABR encephalitis are frequently associated with CSF inflammatory changes, such as pleocytosis and/or the presence of OCB (33–35). In a cohort of 100 patients with anti-NMDAR encephalitis (24) abnormal CSF findings were described in 95% of patients, including CSF pleocytosis in 91%, increased total protein levels in 32% and OCB in 66.7% of anti-NMDAR patients. In a study analyzing clinical data of 44 anti-NMDAR encephalitis patients (36), reported CSF pleocytosis in 68% of patients, OCB was present in altogether 52% of patients during disease course. These findings were also observed in our study, where in anti-LGI1 and anti-Caspr2 positive patients, mainly normal CSF results were found, or increased total protein levels were detected in a few cases. In contrast with previous publications (35), CSF pleocytosis and OCB were exclusively detected in NMDAR patients. Other abnormal CSF findings, such as elevated IgG index and/or increased total protein levels were detected in the GABABR patient group. Data of prevalence of tumor association was also confirmed in our study, where 75% of AE patients presenting with tumor were anti-NMDAR and anti-GABABR positive. Ovarian teratoma is considered the most frequent tumor in anti-NMDAR encephalitis (6), but no association was found in our cohort. In a Chinese series, it was also rare and was only found in 2/72 patients (12). In GABABR encephalitis, the dominant tumor type was small cell lung cancer, occurring in 66.7% of anti-GABABR positive patients, which is in agreement with the findings of Hermetter et al. (6).

Most AE patients in our study showed favorable prognosis. In our cohort, the rate of complete recovery (66.7%) was slightly lower and death rate (10%) was mildly higher compared to data published by Deng et al. (complete recovery: 81.3%, death rate: 6%) (12). In our cohort, relapses occurred exclusively among anti-NMDAR patients (1/19, 5.3% of anti-NMDAR patients). In a cohort of Chinese patients, relapses were also uncommon, occurring in 7/86 (8.1%) AE patients, mainly affecting NMDAR patients (5/72, 6.9% of anti-NMDAR patients) (12). However, in a cohort of Argentine AE patients 25% of anti-NMDAR patients had relapses, but relapses also occurred in 25% of anti-LGI1 patients (15). Majority of the patients received first-line immunotherapy, steroid solely, or in combination with intravenous immunoglobulin or plasmapheresis, which had a good curative effect in most AE patients.

In 63.3% of AE patients in our cohort, signs of inflammation were detected in CSF and/or brain MRI, but no significant correlation was found between inflammatory markers and prognosis. In the study of Escudero et al. (8) a retrospective clinical analysis was conducted in 155 neuronal cell surface autoantibody positive patients with ≥ 60 years of age, but no brain MRI and CSF inflammatory changes were observed. In the cohort of Escudero et al. the most common autoantibody type was anti-LGI1 and the frequency of patients without evidence of CNS inflammation ranged from 25% (LGI1 antibodies) to 7% (GABABR antibodies). Escudero et al. reported higher frequency of non-inflammatory profile in anti-LGI1 patients ≥ 60 years of age (25%), compared to younger patients (age <60 years; 3%). In our study we also reported an overall significantly higher age of onset in AE patients presenting with inflammatory changes. Direct neuronal dysfunction caused by autoantibodies besides inflammatory infiltrates and blood–brain barrier abnormalities may explain this observation (37). Previous publications have found association of CSF changes with worse outcome (38, 39), although early CSF and brain MRI abnormalities did not show a strong correlation with disease outcome (40).

In conclusion, characteristics of AE in our Hungarian multicenter retrospective study are in agreement with previous findings. Anti-NMDAR patients presented with more severe disability at admission compared to anti-LGI1, anti-GABABR and anti-Caspr2 encephalitis. Presence of tumor was associated with worse outcome in AE patients compared to those patients without cancer. However, none of the anti-NMDAR encephalitis female patients had ovarian teratoma. Thirty seven percent of patients lacked presence of both CSF inflammatory markers and brain MRI abnormalities. This observation, in addition to the role of auxiliary examinations (CSF analysis, EEG, brain MRI), emphasizes the importance of clinical presentation and autoantibody testing in diagnostic workflow. Our findings also highlight the significance of early introduction of first-line immunotherapy that resulted in favorable outcome in most AE patients in our cohort.

Our study is limited due to the retrospective data collection performed by clinicians using an online questionnaire, which may result in inadequate accuracy during reporting. The study design precludes the ability to address characteristics of AE that were not directly questioned or consistently recognized (for example, among sleep dysfunctions exclusively the data regarding the presence of insomnia was collected). In our study, due to the low number of pediatric cases with age <10 years (four cases), we could not confidently determine characteristics of pediatric patients. Although, the study has modest sample size, it summarizes detailed clinical data of 35 neuronal surface antibody positive patients from the 60 patients with positive autoantibody test results (N_total_ = 1,034 patients with suspected AE) from 2012 through 2018 in Hungary published in our previous study (10). Our data confirms results of previous publications and further clarifies clinical data of AE patients with neuronal cell surface autoantibodies.

## Data Availability Statement

The original contributions generated for the study are included in the article/Supplementary Material, further inquiries can be directed to the corresponding author/s.

## Ethics Statement

The studies involving human participants were reviewed and approved by Ethics Committee of the Medical Research Council of Hungary (number of approval: 49709-2/2019/EKU). Written informed consent to participate in this study was provided by the participants' legal guardian/next of kin.

## Author Contributions

ZH designed and conceptualized the study, had a major role in the acquisition of data, analyzed the data, and wrote the first draft of the manuscript. BB designed and conceptualized the study and reviewed the manuscript. DS executed the statistical analysis and reviewed the manuscript. GO, MS, FN, TC, ZM, JN, and CR had a major role in acquisition of data and revised the manuscript. ZI and TB designed and conceptualized the study, analyzed the data and revised the manuscript for intellectual content.

## Conflict of Interest

The authors declare that the research was conducted in the absence of any commercial or financial relationships that could be construed as a potential conflict of interest.
