# Supplementary material for: Clinical Characteristics and Outcome of Neuronal Surface Antibody-Mediated Autoimmune Encephalitis Patients in a National Cohort
Source: Front Neurol. 2021 Mar 9;12:611597. doi: 10.3389/fneur.2021.611597 (PMC7985080; doi:10.3389/fneur.2021.611597)
Supplement: Supplementary file 1 [file Presentation_1.PPTX]

## Slide 1
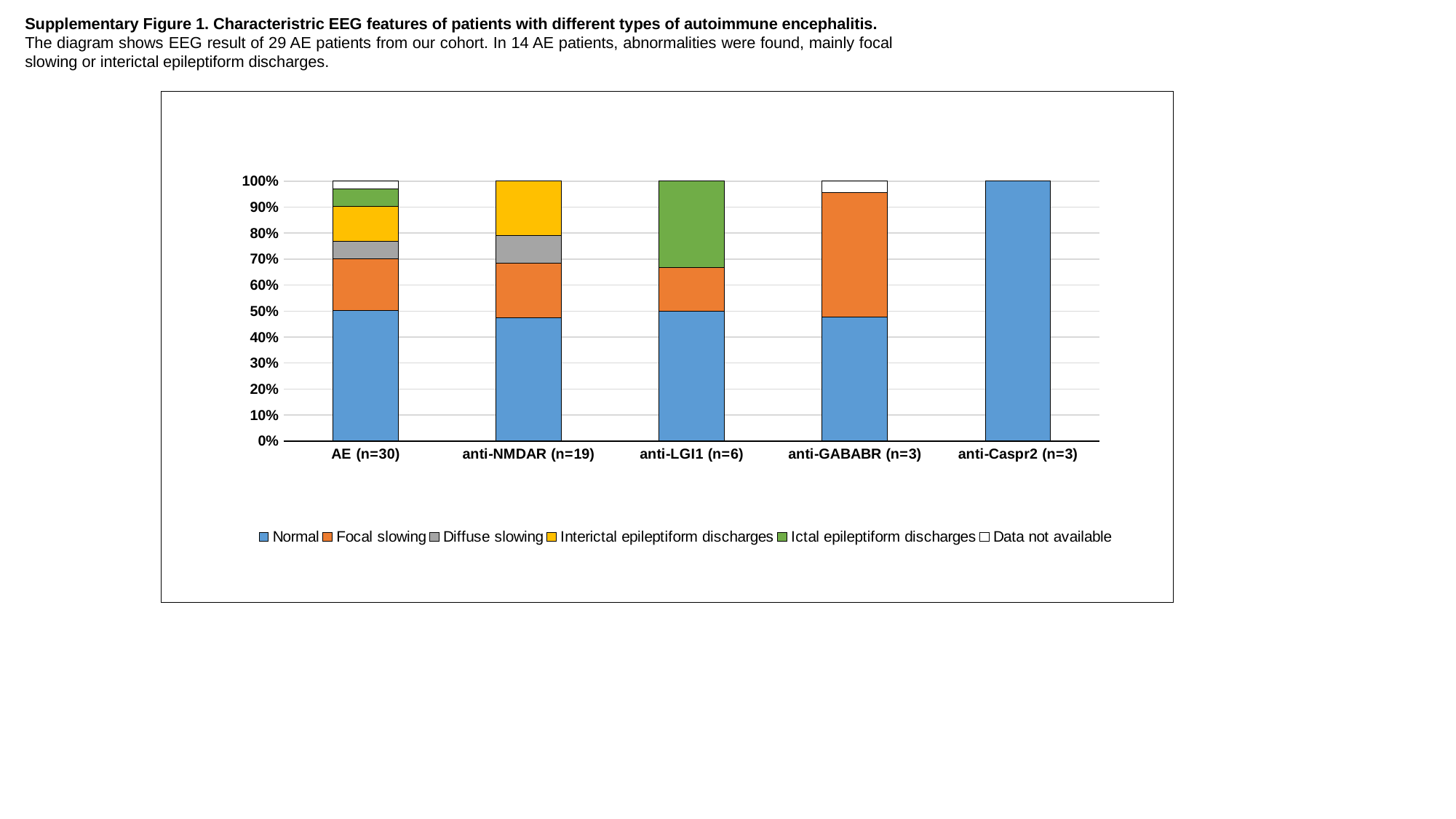

Supplementary Figure 1. Characteristric EEG features of patients with different types of autoimmune encephalitis.
The diagram shows EEG result of 29 AE patients from our cohort. In 14 AE patients, abnormalities were found, mainly focal slowing or interictal epileptiform discharges.
### Chart
| Category | Normal | Focal slowing | Diffuse slowing | Interictal epileptiform discharges | Ictal epileptiform discharges | Data not available |
|---|---|---|---|---|---|---|
| AE (n=30) | 0.5 | 0.2 | 0.067 | 0.133 | 0.067 | 0.03 |
| anti-NMDAR (n=19) | 0.474 | 0.211 | 0.105 | 0.211 | 0.0 | 0.0 |
| anti-LGI1 (n=6) | 0.5 | 0.167 | 0.0 | 0.0 | 0.333 | 0.0 |
| anti-GABABR (n=3) | 0.333 | 0.333 | 0.0 | 0.0 | 0.0 | 0.03 |
| anti-Caspr2 (n=3) | 1.0 | 0.0 | 0.0 | 0.0 | 0.0 | 0.0 |
