# Supplementary material for: Clinical Characteristics and Outcome of Neuronal Surface Antibody-Mediated Autoimmune Encephalitis Patients in a National Cohort
Source: Front Neurol. 2021 Mar 9;12:611597. doi: 10.3389/fneur.2021.611597 (PMC7985080; doi:10.3389/fneur.2021.611597)
Supplement: Supplementary file 2 [file Data_Sheet_1.docx]

**Supplementary Table 1: Prodrome factors and preceding HSV infection in AE patients.**

|  | **Autoimmune encephalitis** | **anti-NMDAR** | **anti-LGI1** | **anti-GABABR** | **anti-Caspr2** |
| --- | --- | --- | --- | --- | --- |
|  | **(n=30)** | **(n=19)** | **(n=6)** | **(n=3)** | **(n=3)** |
| **Fever/flu-like symptoms (n, %)** | 10 (33.3%) | 8 (42.1%) | 1 (16.7%) | 1 (33.3%) | 0 |
| **Headache (n, %)** | 3 (10%) | 2 (10.5%) | 0 | 1 (33.3%) | 0 |
| **Vomiting (n, %)** | 1 (3.3%) | 1 (5.3%) | 0 | 0 | 0 |
| **HSV infection (n, %)** | 1 (3.3%) | 1 (5.3%) | 0 | 0 | 0 |
| **Systemic HSV IgM positivity (n, %)** | 4 (13.3%) | 4 (21.5%) | 0 | 0 | 0 |

*NMDAR: N-methyl-D-aspartate receptor; LGI1: leucine-rich, glioma inactivated 1; GABABR: γ-aminobutyric acid receptor-B; Caspr2: contactin-associated protein-like 2; HSV: herpes simplex virus.*

| **Case number** | **Age** | **Sex (M/F)** | **Antibody** | **Clinical presentation*** | **CSF** | **MRI** | **EEG** | **Time to diagnosis (months)** | **mRS (diagnosis)** | **mRS**  **(last visit)** |
| --- | --- | --- | --- | --- | --- | --- | --- | --- | --- | --- |
| 1 | 24 | M | NMDAR | Psychiatric symptoms, memory loss | OCB | BTH | Focal slowing | 1 | 4 | 3 |
| 2 | 21 | M | NMDAR | Psychiatric symptoms, insomnia, dyskinesias, seizures, memory loss | OCB+IgG index↑ | BTH | Focal slowing | 2 | 5 | 0 |
| 3 | 20 | F | NMDAR | Psychiatric symptoms, seizures, memory loss | NL | NL | NL | 4 | 3 | 0 |
| ♦ 4 | 23 | M | NMDAR | Seizures, aphasia, memory loss | Pleo+TP↑ | UTH | IEDs | 2 | 2 | 0 |
| 5 | 32 | M | NMDAR | Seizures | - | Unilateral hippocampal sclerosis | NL | 2 | 2 | 0 |
| 6 | 70 | F | NMDAR | Psychiatric symptoms, memory loss | NL | NL | NL | 1 | 5 | 6 |
| 7 | 55 | M | NMDAR | Memory loss, psychiatric symptoms | Pleo+TP↑ | UTH | NL | 3 | 3 | 2 |
| 8 | 75 | F | NMDAR | Hyponatraemia, seizures, dyskinesias, psychiatric symptoms | TP↑ | BTH | IEDs | 1 | 3 | 0 |
| 9 | 48 | M | NMDAR | Seizures, piloerection, hyponatraemia, psychiatric symptoms | OCB+TP↑ | NL | NL | 9 | 5 | 0 |
| 10 | 16 | F | NMDAR | Psychiatric symptoms, seizures, dyskinesias, dysarthria | OCB | NL | NL | 1 | 5 | 0 |
| 11 | 17 | M | NMDAR | Seizures, status epilepticus, psychiatric symptoms, dyskinesias | NL | - | Diffuse slowing | 1 | 5 | 1 |
| 12 | 17 | F | NMDAR | Psychiatric symptoms, seizures, insomnia | NL | NL | NL | 1 | 4 | 0 |
| 13 | 1 | F | NMDAR | Psychiatric symptoms, seizures, ataxia, choreoathetosis, dyskinesias | NL | NL | NL | 1 | 5 | 0 |
| 14 | 5 | M | NMDAR | Ataxia, dysarthria, seizures, psychiatric symptoms, dyskineasias, dysautonomia | - | NL | Focal slowing | 1 | 5 | 0 |
| 15 | 21 | M | NMDAR | Psychiatric symptoms, memory loss, ataxia, cerebellar symptoms, neuropathy | - | Unilateral extratemporal hyperintensity | NL | - | 5 | 0 |
| 16 | 29 | F | NMDAR | Psychiatric symptoms, seizures, status epilepticus, dyskinesias, dystonia, memory loss | NL | NL | Diffuse slowing | - | 5 | 0 |
| 17 | 70 | M | NMDAR | Psychiatric symptoms, seizures | - | NL | Focal slowing | - | 3 | 0 |
| 18 | 9 | F | NMDAR | Psychiatric symptoms, seizures, status epilepticus, aphasia, dystonia | NL | NL | IEDs | - | 5 | 0 |
| 19 | 65 | M | NMDAR | Psychiatric symptoms, left central facial lesion, increased tone in extremities, right limb hypotonia | OCB | NL | IEDs | - | 5 | 2 |
| 20 | 65 | M | LGI1 | Psychiatric symptoms, hyponatraemia, FBDS, seizures, memory loss | NL | UTH | NL | 5 | 3 | 0 |
| 21 | 54 | M | LGI1 | Psychiatric symptoms, seizures, hyponatraemia, memory loss | NL | BTH | Ictal epileptiform discharges | 53 | 3 | 2 |
| 22 | 51 | M | LGI1 | Memory loss, seizures, FBDS, hyponatraemia, insomnia, psychiatric symptoms | TP↑ | NL | Ictal epileptiform discharges | 36 | 3 | 0 |
| 23 | 58 | M | LGI1 | FBDS, hyponatraemia, dysautonomia, insomnia | TP↑ | NL | NL | 1 | 3 | 0 |
| 24 | 50 | F | LGI1 | Memory loss | NL | BTH | Focal slowing | 1 | 3 | 6 |
| 25 | 67 | M | GABABR | Psychiatric symptoms, memory loss | NL | UTH | NL | 3 | 3 | 6 |
| 26 | 16 | F | GABABR | Dyskinesias, memory loss, insomnia | TP↑ | - | - | 24 | 2 | 1 |
| 27 | 58 | M | GABABR | Psychiatric symptoms, seizures, hyponatraemia | TP+IgG index↑ | UTH | Focal slowing | 7 | 5 | 4 |
| 28 | 72 | M | Caspr2 | Seizures, psychiatric symptoms, memory loss | TP↑ | UTH | NL | 1 | 3 | 0 |
| 29 | 68 | F | Caspr2 | Psychiatric symptoms, aphasia, seizures, status epilepticus | - | UTH | NL | 9 | 4 | 0 |
| 30 | 3 | M | LGI1+Caspr2 | Psychiatric symptoms, seizures, hyponatraemia, insomnia, skin rashes | - | - | NL | 1 | 4 | 0 |

**Supplementary Table 2: Clinical manifestations, auxiliary examinations and follow-up data of AE patients**

*(*in order of onset of symptoms; ♦ HSV encephalitis preceded development of secunder anti-NMDAR encephalitis)*

*M: male; F: female; NMDAR: N-methyl-D-aspartate receptor; LGI1: leucine-rich, glioma inactivated 1; GABABR: γ-aminobutyric acid receptor-B; Caspr2: contactin-associated protein-like 2; FBDS: faciobrachial dystonic seizures; CSF: cerebrospinal fluid; NL: normal; OCB: oligoclonal band; Pleo: pleocytosis; TP: total protein; UTH: unilateral temporal hyperintensity; BTH: bilateral temporal hyperintensities; IEDs: interictal epileptiform discharge; mRS: modified Rankin scale score.*

**Supplementary Table 3: Associated tumors in patients with AE**

| **Antibody** | **Age**  **(years)** | **Sex (M/F)** | **Tumor type** | **Tumor detection (months)*** | **Treatment** | **Outcome** |
| --- | --- | --- | --- | --- | --- | --- |
| NMDAR | 70 | F | LCNEC | 87 (prior) | Tumor resection, chemotherapy, radiotherapy | Death |
| NMDAR | 21 | M | Chondroblastoma | 33 (after) | Tumor resection | Complete remission |
| NMDAR | 70 | M | Prostate cancer | 80 (after) | Radiotherapy | No remission |
| GABABR | 76 | M | SCLC | 15 (after) | Chemotherapy | Death |
| GABABR | 16 | F | Thymoma | 5 (after) | Treatment refusal | No remission |
| GABABR | 58 | M | SCLC | 1 (after) | Chemotherapy | No remission |
| LGI1 | 50 | F | Meningeal MALT-lymphoma | 78 (prior) | Tumor resection, chemotherapy, radiotherapy | Complete remission |
| Caspr2 | 72 | M | DLBCL | 23 (prior) | Chemotherapy | No remission |

*NMDAR: N-methyl-D-aspartate receptor; LGI1: leucine-rich, glioma inactivated 1; GABABR: γ-aminobutyric acid receptor-B; Caspr2: contactin-associated protein-like 2; LCNEC: large cell neuroendocrine carcinoma; DLBCL: diffuse large B-cell lymphoma.*

** compared to the time of positive neuronal autoantibody test result*

**Supplementary Table 4: EEG data of AE patients.**

|  | **Autoimmune encephalitis** | **anti-NMDAR** | **anti-LGI1** | **anti-GABABR** | **anti-Caspr2** |
| --- | --- | --- | --- | --- | --- |
|  | **(n=30)** | **(n=19)** | **(n=6)** | **(n=3)** | **(n=3)** |
| **Data not available** | 1 | 0 | 0 | 1 (33.3%) | 0 |
| **Normal** | 15 | 9 (47.4%) | 3 (50%) | 1 (33.3%) | 3 (100%) |
| **Focal slowing** | 6 | 4 (21.1%) | 1 (16.7%) | 1 (33.3%) | 0 |
| **Diffuse slowing** | 2 | 2 (10.5%) | 0 | 0 | 0 |
| **Interictal epileptiform discharges** | 4 | 4 (21.1%) | 0 | 0 | 0 |
| **Ictal epileptiform discharges** | 2 | 0 | 2 (33.3%) | 0 | 0 |

*NMDAR: N-methyl-D-aspartate receptor; LGI1: leucine-rich, glioma inactivated 1; GABABR: γ-aminobutyric acid receptor-B; Caspr2: contactin-associated protein-like 2.*

**Supplementary Table 5: Brain MRI data of AE patients**

|  | **Autoimmune encephalitis** | **anti-NMDAR** | **anti-LGI1** | **anti-GABABR** | **anti-Caspr2** |
| --- | --- | --- | --- | --- | --- |
|  | **(n=30)** | **(n=19)** | **(n=6)** | **(n=3)** | **(n=3)** |
| **Data not available** | 3 | 1 | 1 | 1 | 1 |
| **Normal** | 13 | 11 | 2 | 0 | 0 |
| **Unilateral temporal hyperintensity (contrast enhancement)** | 7 | 2 | 1 | 2 | 2 |
| **Bilateral temporal hyperintensities (contrast enhancement)** | 5 | 3 | 2 | 0 | 0 |
| **Unilateral hippocampal sclerosis (contrast enhancement)** | 1 | 1 | 0 | 0 | 0 |
| **Unilateral extratemporal hyperintensity (no contrast enhancement)** | 1 | 1 | 0 | 0 | 0 |

*NMDAR: N-methyl-D-aspartate receptor; LGI1: leucine-rich, glioma inactivated 1; GABABR: γ-aminobutyric acid receptor-B; Caspr2: contactin-associated protein-like 2.*

**Supplementary Table 6: Immunotherapy of AE patients**

|  | **Autoimmune encephalitis** | **anti-NMDAR** | **anti-LGI1** | **anti-GABABR** | **anti-Caspr2** |
| --- | --- | --- | --- | --- | --- |
|  | **(n=30)** | **(n=19)** | **(n=6)** | **(n=3)** | **(n=3)** |
| **First-line therapy** | 24 (80%) | 14 (73.7%) | 6 (100%) | 2 (66.7%) | 3 (100%) |
| Only steroid | 4 (13.3%) | 3 (15.8%) | 1 (16.7%) | 0 | 0 |
| Only PE | 1 (3.3%) | 0 | 0 | 0 | 1 (33.3%) |
| Steroid+IVIG | 3 (10%) | 1 (5.3%) | 1 (16.7%) | 0 | 2 (66.7%) |
| Steroid+PE | 12 (40%) | 7 (36.8%) | 3 (50%) | 2 (66.7%) | 0 |
| Steroid+IVIG+PE | 4 (13.3%) | 3 (15.8%) | 1 (16.7%) | 0 | 0 |
| **Second-line therapy** | 4 (13.3%) | 4 (21.1%) | 0 | 0 | 0 |
| Azathioprine | 1 (3.3%) | 1 (5.3%) | 0 | 0 | 0 |
| Rituximab | 1 (3.3%) | 1 (5.3%) | 0 | 0 | 0 |
| Azathioprine+Rituximab | 2 (6.7%) | 2 (10.5%) | 0 | 0 | 0 |

*NMDAR: N-methyl-D-aspartate receptor; LGI1: leucine-rich, glioma inactivated 1; GABABR: γ-aminobutyric acid receptor-B; Caspr2: contactin-associated protein-like 2; PE: plasmapheresis; IVIG: intravenous immunoglobulin.*
